# Developmental Pb exposure increases AD risk *via* altered intracellular Ca^2+^ homeostasis in hiPSC-derived cortical neurons

**DOI:** 10.1016/j.jbc.2023.105023

**Published:** 2023-07-07

**Authors:** Junkai Xie, Shichen Wu, Hailey Szadowski, Sehong Min, Yang Yang, Aaron B. Bowman, Jean-Christophe Rochet, Jennifer L. Freeman, Chongli Yuan

**Affiliations:** 1 Davidson School of Chemical Engineering, Purdue University, West Lafayette, Indiana, USA; 2 Agriculture and Biological Engineering, Purdue University, West Lafayette, Indiana, USA; 3 Department of Medicinal Chemistry and Molecular Pharmacy, Purdue University, West Lafayette, Indiana, USA; 4 Purdue Institute of Integrated Neuroscience, Purdue University, West Lafayette, Indiana, USA; 5 School of Health Sciences, Purdue University, West Lafayette, Indiana, USA; 6 Purdue Center of Cancer Research, Purdue University, West Lafayette, Indiana, USA

**Keywords:** Pb exposure, hiPSC, cortical neuron, neurodegenerative disease, Alzheimer’s disease

## Abstract

Exposure to environmental chemicals such as lead (Pb) during vulnerable developmental periods can result in adverse health outcomes later in life. Human cohort studies have demonstrated associations between developmental Pb exposure and Alzheimer’s disease (AD) onset in later life which were further corroborated by findings from animal studies. The molecular pathway linking developmental Pb exposure and increased AD risk, however, remains elusive. In this work, we used human iPSC-derived cortical neurons as a model system to study the effects of Pb exposure on AD-like pathogenesis in human cortical neurons. We exposed neural progenitor cells derived from human iPSC to 0, 15, and 50 ppb Pb for 48 h, removed Pb-containing medium, and further differentiated them into cortical neurons. Immunofluorescence, Western blotting, RNA-sequencing, ELISA, and FRET reporter cell lines were used to determine changes in AD-like pathogenesis in differentiated cortical neurons. Exposing neural progenitor cells to low-dose Pb, mimicking a developmental exposure, can result in altered neurite morphology. Differentiated neurons exhibit altered calcium homeostasis, synaptic plasticity, and epigenetic landscape along with elevated AD-like pathogenesis markers, including phosphorylated tau, tau aggregates, and Aβ42/40. Collectively, our findings provide an evidence base for Ca dysregulation caused by developmental Pb exposure as a plausible molecular mechanism accounting for increased AD risk in populations with developmental Pb exposure.

Lead (Pb) is a heavy metal that persists in the environment and has been considered a major public health concern due to its legacy uses in the industry (1). Although Pb is highly regulated by the US Environmental Protection Agency (EPA), a significant fraction of the population is still exposed to Pb through the consumption of contaminated food and/or water (2). In 2016, the Centers for Disease Control (CDC) reported that the average blood lead level in the US population is 0.920 μg/dl (3) below the current blood Pb reference value, which is 5 and 3.5 μg/dl for adults and children set by the CDC, respectively (4, 5). Exposure to high levels of Pb is associated with acute symptoms, such as headaches, restlessness, and abdominal cramps (6), and is more frequently observed in children below the age of three. Chronic exposure to low concentrations of Pb is more common among the population and has a strong link to several long-term adverse health effects, including hypertension, renal failure, and most recently increased risks of late-onset neurological diseases, notably Alzheimer’s disease (AD) and Parkinson’s disease (7, 8).

The developmental period, including prenatal and perinatal, is a highly sensitive window toward exposure that contributes to shaping health status later in life based on the developmental origin of the health and disease paradigm. Clinical outcomes of low-dose Pb exposure in infants and children include declines in learning ability and visual-motor coordination, as well as increases in distractibility, reaction times, and antisocial behavior (9, 10). Children exposed to Pb also suffer from neurodevelopmental, psychiatric, and behavioral disorders (11, 12). Longitudinal studies show these cognitive effects of developmental Pb exposure persist into adulthood (13, 14, 15). Furthermore, increasing literature evidence suggests that chronic low-level Pb exposure is associated with an increased risk of neurodegenerative diseases (7, 8). The connection between developmental Pb exposure and neurodegeneration, particularly AD, has been observed in various animal models, including mice (16, 17), rat (18), zebrafish (19), and monkeys (20). The molecular mechanism that remains latent over years and contributes to AD onset, however, remains elusive.

Among multiple neurotoxic mechanisms, one of the prevailing hypotheses suggests that the neurotoxicity of Pb arises from its similarity to Ca which plays significant roles in neuronal signaling based on valency and molecular size (21). Specifically, Pb competes with intracellular Ca to bind to multiple proteins essential for miscellaneous signaling pathways, including the binding of calcium to synaptotagmin I (22) and the activation of calmodulin (23). Specifically, calmodulin (CaM), a key Ca-activated protein, has a higher binding affinity for Pb than Ca (24). Furthermore, Pb also disrupts neurotransmission by interfering with voltage-gated Ca channels (25) and acting as a noncompetitive antagonist of postsynaptic N-Methyl-D-aspartate (NMDA) receptors, a glutamatergic receptor heavily involved in learning and memory (26). Disrupted signal transduction pathways have been observed to negatively impact the plasticity and long-term potentiation (LTP) of neurons in animal models. Studies on the molecular impact of low-dose, early developmental Pb exposure in rats and zebrafish reported reduced neural plasticity and impaired LTP of neurons by increasing oxidative stress, decreasing neurogenesis, and interfering with neurotransmission (27, 28, 29, 30).

Although the mechanisms outlined earlier partially account for Pb-induced neurotoxicity arising from developmental exposure, the long latent period between exposure events and disease on-set remains largely unaddressed. The transgenerational Pb-exposure effects observed in mouse models (31, 32) further verified the involvement of epigenetic mechanisms as an underlying molecular mechanism transmitting late-onset disease risks. Epigenetic modifications, including 5 mC and H3K27me3, have been identified as potential biomarkers indicating the onset of neurodevelopmental (33, 34) and neurodegenerative diseases (35). Epigenetic modifications, particularly DNA and histone methylation, can be maintained for an extended period and have the potential to be passed down to offspring. We thus expect that exposure to environmental insults causing significant differences in DNA and histone methylation during early development can have a variety of implications for future cell lineages. Literature reports a decreased amount of DNA methylation following Pb exposure in human (36), rodent (37), and zebrafish (38) models. Prior work from our group examined the potential persistence of epigenetic modifications in human cell lines, namely, HEK293 and SH-SY5Y cells, and found that exposure to Pb led to persistent change in 5 mC level, which was correlated with cell development (39, 40). Human-induced pluripotent stem cells (hiPSCs) have gained increased popularity as an *in vitro* platform for studying brain biology. Cortical neurons with a high percentage of glutamatergic neurons resembling the critical brain region involved in AD, namely, cerebral cortex (41) and hippocampus (42), can be differentiated from hiPSC following well-established protocols (43, 44) offering unprecedented opportunities to evaluate the impact of developmental Pb exposure on neuron formation and functionality.

Here, we evaluated the effect of prior developmental Pb exposure on neuronal differentiation, synapse formation, epigenetic markers, the transcriptome, Ca homeostasis, and AD-related pathological changes. We observed persistent alterations in genes regulating Ca homeostasis and elevations in AD hallmarks, including phosphorylated tau in treated neurons as well as tau aggregates and the β-amyloid peptide variant Aβ42 in the cell culture medium. Based on the collective findings, a plausible mechanism was proposed linking developmental Pb exposure and AD risks later in life.

## Results

### Pb exposure results in persistent changes in neuron development and morphology

**Figure 1:**
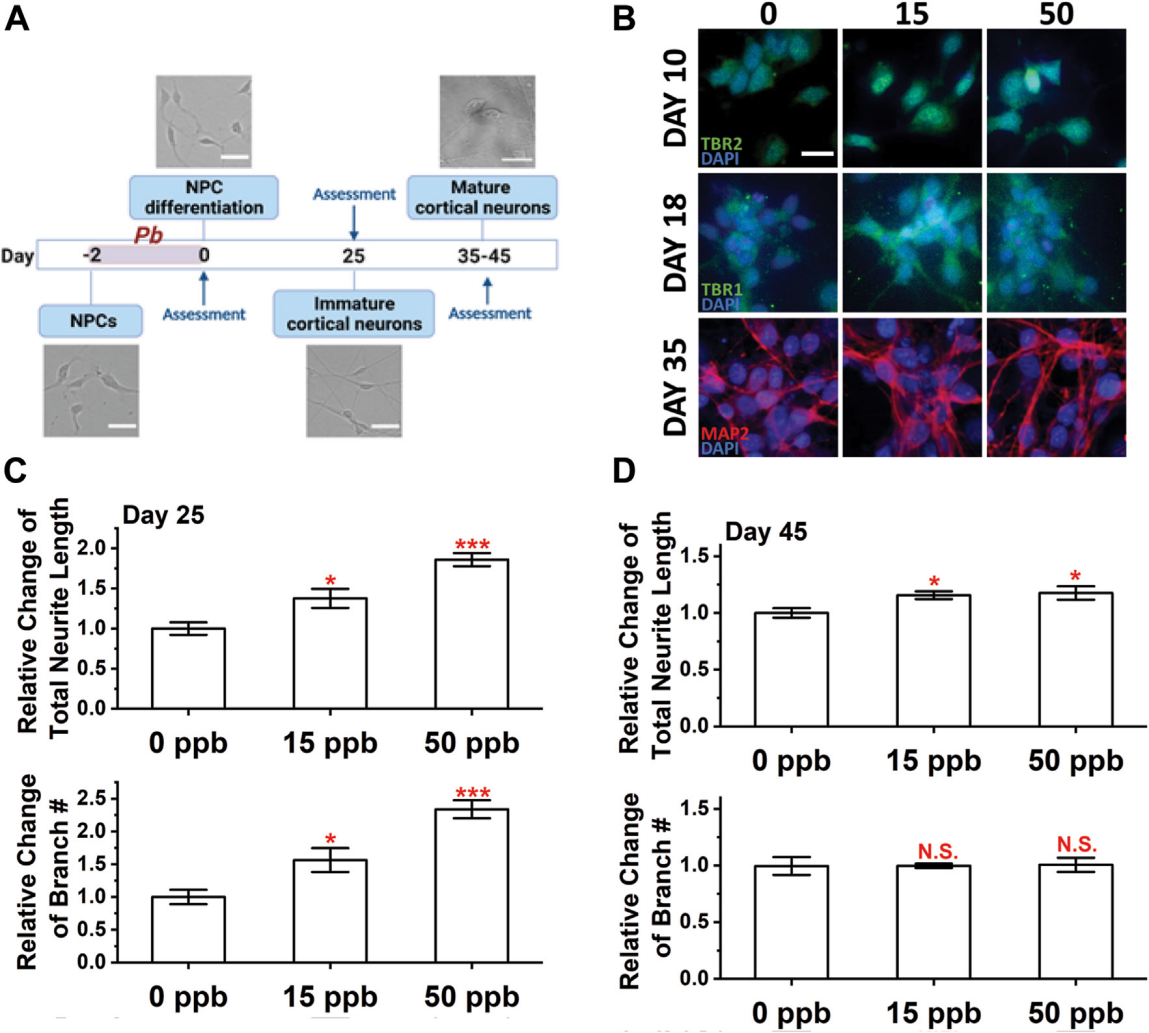
**Alterations in neural morphology after developmental exposure to Pb.***A*, illustration of the exposure, differentiation, and assessment scheme for this work. *B*, representative images of differentiating cortical neurons at Day 10, 18, and 35. TBR2 stains for intermediate progenitor cells, TBR1 stains for immature neurons, and MAP2 stains for mature neurons. Scale bar = 20 μm. Total neurite length (*top*) and branch number (*bottom*) were quantified at (*C*) Day 25 and (*D*) Day 45. N ≥ 10 views from three independent differentiations. Data = mean ± SE. N.S., not significant; ∗*p* < 0.05 and ∗∗∗*p* < 0.001.

Following an established protocol (44), we differentiated hiPSCs into neural progenitor cells (NPCs) identified with PAX6 and FOXG1, two common markers of NPCs (45, 46) (Fig. S1). To assess the effects of Pb exposure on nervous system development, we exposed NPCs to 0, 15, and 50 ppb Pb for 48 h before proceeding to differentiation as summarized in Figure 1*A*. The dose of Pb exposure was selected based on the regulation standards and prevailing Pb concentrations found in the environment. Specifically, 15 ppb (1.5 μg/dl) is the maximum concentration allowed in drinking water according to the US EPA (47). In all, 50 ppb (5 μg/dl) was the blood lead reference level for children set by the CDC, which was further lowered to 35 ppb (3.5 μg/dl) in 2021 (48). In all, 15 and 50 ppb Pb exposure does not alter cell viability (Fig. S2*A*) or density (Fig. S2*B*) and is thus suitable for evaluating long-term neurotoxicity. NPCs are abundant in developing brains, particularly during the second (49) and third (50) trimesters. We thus exposed NPC to defined doses of Pb mimicking a developmental exposure.

To start, we assessed how prior Pb exposure may affect the differentiation process using cell markers for intermediate progenitor cells (TBR2), immature neurons (TBR1), and mature neurons (MAP2). Differentiating cells were stained for these markers at selected time points as summarized in Figure 1*B*. Most of the cells were found to be TBR2+, TBR1+, and MAP2+ on Days 10, 18, and 35, respectively, with no significant difference among cultures exposed to different Pb doses. We further verified the identity and composition of cortical neuron cultures on Day 35 using the markers VGLUT1 and GFAP as shown in Fig. S3. Differentiated neurons were almost all positive with VGLUT1 regardless of Pb exposure, and a few GFAP astrocytes were found in any of the cultures.

We then performed neurite outgrowth analysis on immature (Day 25) and mature (Day 45) neurons as shown in Figure 1, *C* and *D*, respectively. Immature neurons with developmental exposure to Pb had significantly longer neurite lengths (an increase of ∼40% and 85% compared to the control for 15 and 50 ppb of Pb, respectively). A similar trend was observed for the number of branches on immature (Day 25) neurons. Mature neurons at Day 45 still had increased neurite lengths after Pb exposure, but the difference among cultures exposed to varying Pb doses diminished. No difference was detected in the number of branches among cultures exposed to different Pb concentrations.

### Persistence of the epigenetic landscape alterations after developmental Pb exposure

We then assessed nuclear morphology and chromatin compactness. Nuclear area and roundness were measured using DAPI-stained nuclei from Day 25 and Day 45 following our established protocol (39, 51, 52, 53). The data were summarized in Fig. S4, *A* and *B* for Days 25 and 45, respectively. Developmental exposure to Pb did not affect the nuclear area, but slightly altered nuclear roundness after exposure to 50 ppb Pb on Day 25 suggesting a more anisotropic nuclear shape. The trend persists on Day 45. No changes were detected in nuclear compactness at either Day 25 or 45.

**Figure 2:**
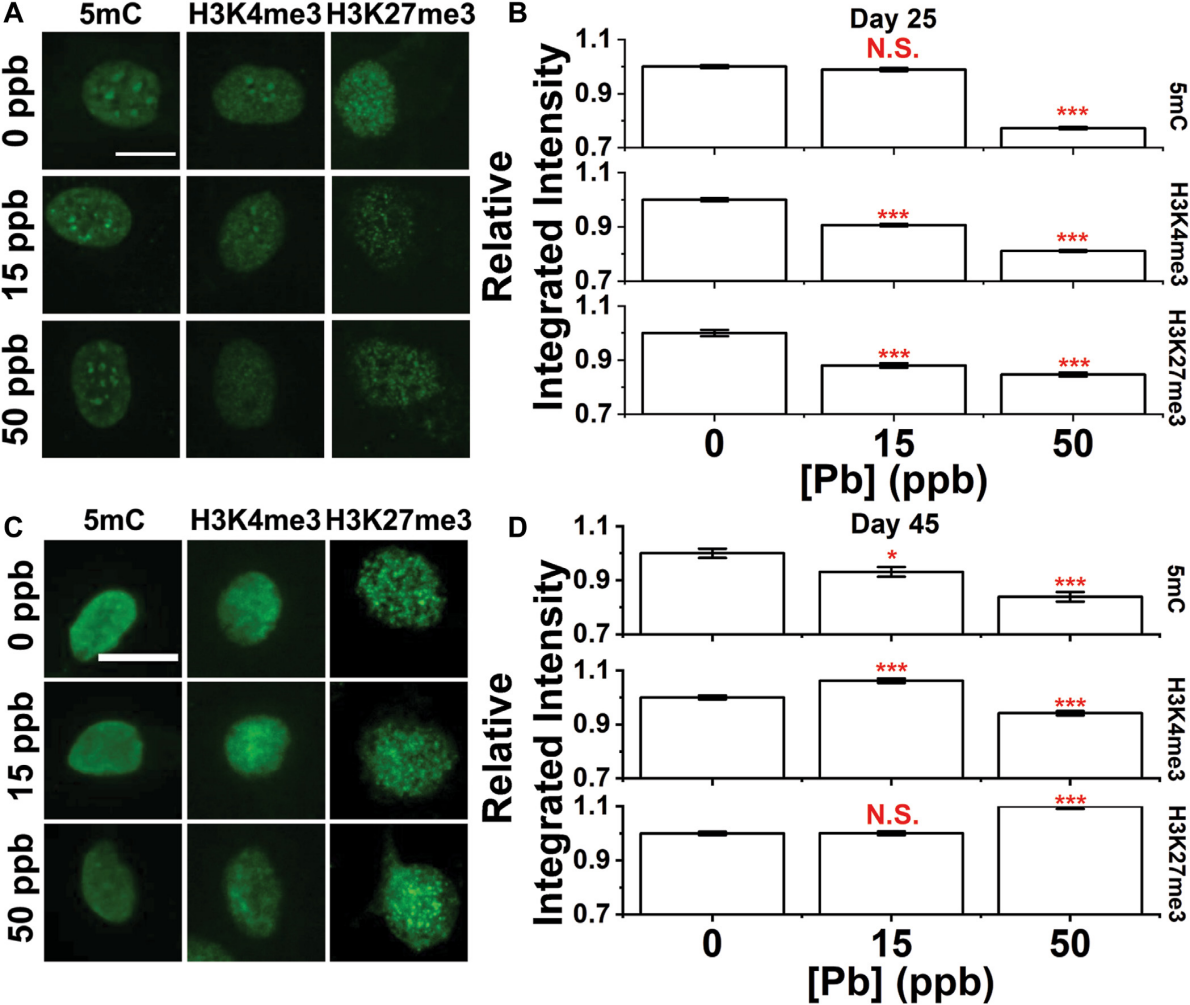
**Persistant alterations in epigenetic landscape of differentiated neurons after developemental Pb exposure.** Image panel of neurons at (*A*) Day 25 and (*B*) Day 45 stained for 5 mC, H3K4me3, and H3K27me3 markers. Scale bar = 10 μm. Relative change in integrated intensity of 5 mC, H3K4me3, and H3K27me3 markers of neurons at (*C*) Day 25 and (*D*) Day 45. Data = Mean ± SE. N > 600 cells from six independent differentiations. N.S., not significant; ∗*p* < 0.05 and ∗∗∗*p* < 0.001.

We characterized changes in three epigenetic markers, namely cytosine methylation (5 mC), H3K4me3, and H3K27me3, all of which are characteristic markers of repressed chromatin regions (54, 55, 56, 57). Typical images of stained cells in Day 25 and 45 cultures are summarized in Figure 2, *A* and *C*. At Day 25, we observed significant decreases in all three markers with changes more significant at a higher dose of Pb. Changes in H3K4me3 and H3K27me3 were compensated on Day 45. A decrease in 5 mC, however, was found to persist with significant changes in neurons exposed to both 15 and 50 ppb Pb at Day 45. Changes in epigenetic markers were further verified using ELISA for 5 mC, and Western blot for H3K27me3 and H3K9me3 with results summarized in Fig. S4, *C*–*H*. Similar trends were observed on both days with compensatory effects over time.

### Transcriptomic analysis of neuron culture after developmental Pb exposure

**Figure 3:**
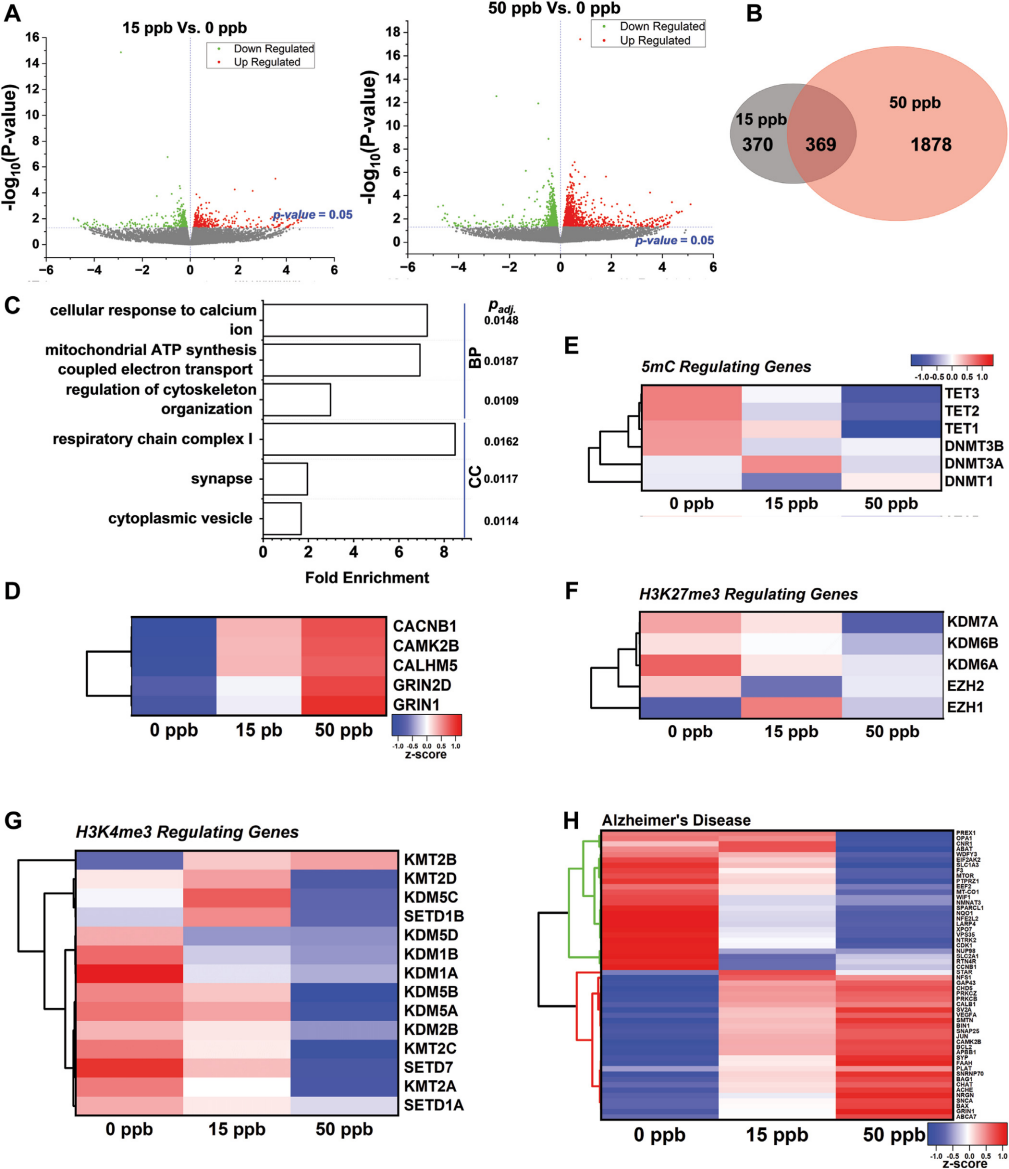
**Transcriptomic analysis of Pb-treated neuronal culture.***A*, volcano plots of DEGs identified for mature neurons (Day 45) of (*left*) 15 ppb vs. 0 ppb and (*right*) 50 ppb vs. 0 ppb of prior Pb treatment. N = 3 independent differentiations. *B*, Venn diagram summarized the shared DEGs between 15 ppb and 50 ppb. *C*, *top* biological pathways (BP) and cellular compartments (CC) altered by Pb exposure *via* Gene Ontology analysis with P_adj_ next to each pathway. Standardized mean transcription level of genes regulating (*D*) calcium homeostasis, (*E*) 5 mC, (*F*) H3K27me3, (*G*) H3K4me3, and (*H*) genes associated with Alzheimer’s Disease. The heat maps in (*E*–*G*) share the same look-up table (LUT). Normalized gene expression was represented as z-score where z-score = ((gene expression of each group) − (mean expression across all groups))/(standard deviation of gene expression across all groups).

With persistent Pb-dependent changes in the epigenetic landscape, particularly 5 mC, we proceeded to evaluate transcriptomic changes in neurons *via* RNA-sequencing (RNA-seq). Volcano plots showing the number and fold changes of differentially expressed genes (DEGs) identified for 0 to 15 ppb and 0 to 50 ppb are summarized in Figure 3*A*. The similarity of DEGs from 15 and 50 ppb Pb-treated cells is summarized *via* a Venn diagram in Figure 3*B*. We performed gene enrichment analysis *via* Gene Ontology (GO) using Pb-untreated and treated samples (see Table S1); and the top enriched biological processes (BP) and cellular compartments (CC) are shown in Figure 3*C*. From all enriched biological processes, the cellular response to calcium emerged as the top altered process with more than seven-fold DEG enrichments (*p*_*adj*_ = 0.0148). Mitochondrial ATP synthesis and cytoskeleton organization were also noticeably altered. The top affected cellular compartment was found to be respiratory chain complex I, associated with mitochondrial ATP synthesis. The synapse, a major compartment for calcium intake, and cytoplasmic vesicles that participate in multiple neuronal signaling and cell trafficking events were ranked second and third among affected cellular compartments.

We identified five genes that were consistently altered after in cultures exposed to 15 and 50 ppb Pb and are known to contribute to neuronal calcium homeostasis; their mean transcriptional levels are shown in Figure 3*D*. Among them, the voltage-gated calcium channel *CACNB1* (15 ppb: 13%; 50 ppb: 19%) and ionotropic glutamate receptor *GRIN1* (15 ppb: 11%; 50 ppb: 23%) were significantly upregulated. *CaMK2B*, a calcium-regulated kinase, was also significantly upregulated (15 ppb: 23%; 50 ppb: 28%).

We also evaluated changes in epigenetic enzymes regulating the selected epigenetic modifications, namely, 5 mC, H3K27me3, and H3K4me3 as shown in Figure 3, *E*–*G*. Among them, *TET1* and *TET2* were significantly downregulated in the cultures exposed to 50 ppb Pb (*TET1*: −22.5%; *TET2*: −15.5%). The expression of DNA methyltransferases including *DNMT1, DNMT3A*, and *DNMT3B*, however, was not significantly altered. No significant changes were observed for genes regulating H3K27me3. *KMT2B* and *KMT2C*, the methyltransferases, involved in converting H3K4 to the H3K4me3 form, were significantly altered in cultures treated with 50 ppb Pb (*KMT2B*: +12% and *KMT2C*: −13.5%) and the H3K4me3 demethylase, *KDM5A*, showed significant down-regulation (−14.7%) under the same exposure conditions.

Furthermore, we identified AD-associated gene sets that were altered after Pb exposure at both doses summarized in Figure 3*H* and Table S2. DEGs were clustered based on transcriptional levels and changes in the expression levels of these genes exhibited a clear association with Pb doses.

### Developmental Pb exposure alters synapse density and Ca homeostasis

**Figure 4:**
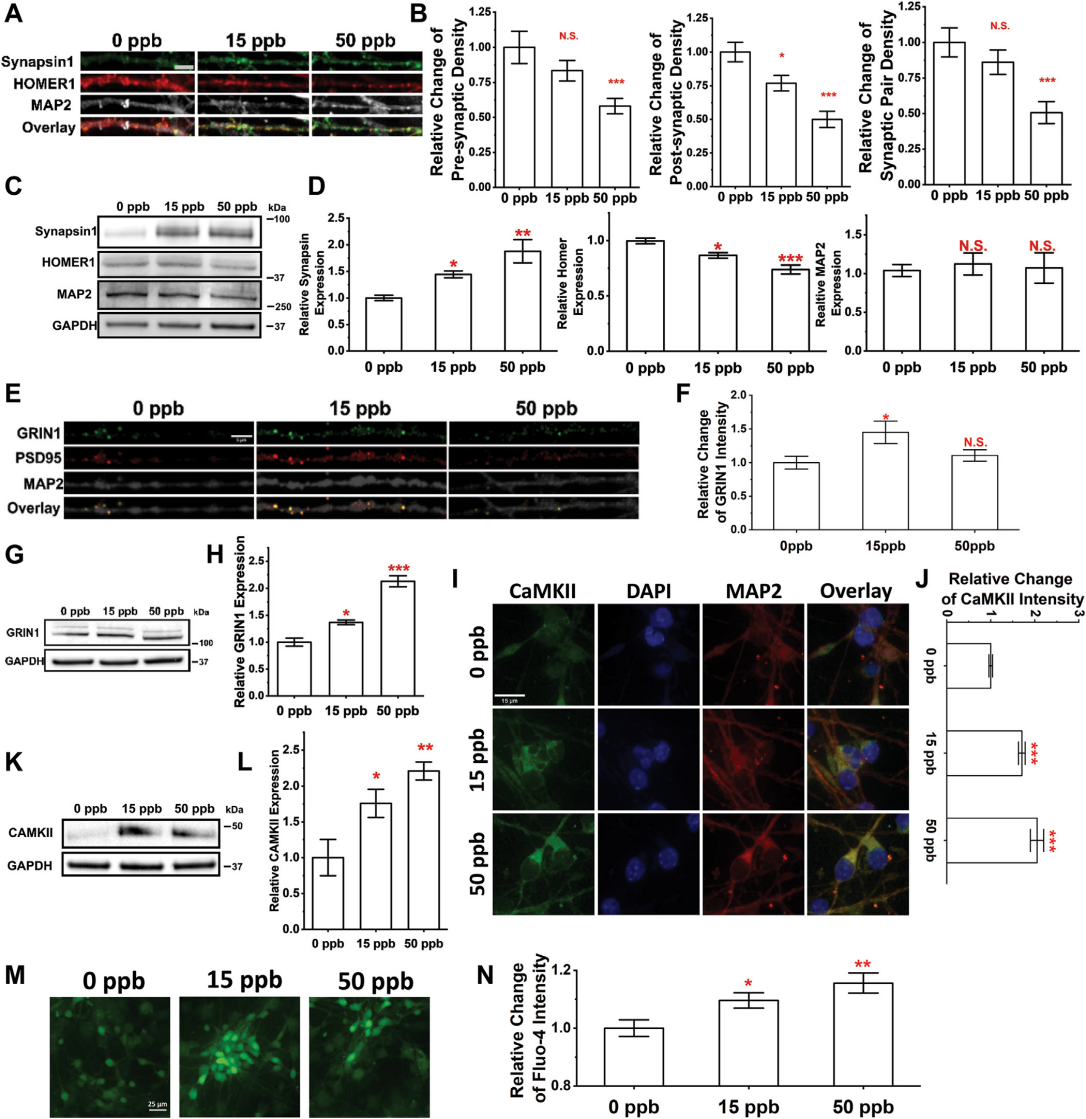
**Developmental exposure to Pb leads to changes in synaptic density and calcium homeostasis.***A*, representative confocal images of mature neurons stained for DAPI, MAP2, Synapsin1/2, and HOMER1 at Day 45. Scale bar = 10 μm. *B*, relative changes in pre-synapse (*left*), post-synapse (*middle*), and synaptic pair (right) density of mature neurons after Pb exposure. N ≥ 15 neurites from three independent differentiations. *C*, typical Western blot images examining Synapsin1, HOMER1, and MAP2 expression with their relative expression level quantified in (*D*) using protein extracts of cortical neuron cultures at Day 45. N = 3 independent differentiations. *E*, confocal images of neurites stained for GRIN1, PSD95 and MAP2. Scale bar = 5 μm. *F*, a summary of relative changes in GRIN1 intensity in PSD95^+^/GRIN1^+^ post-synapses after developmental exposure to varying doses of Pb. N ≥ 10 neurites from two independent differentiations. *G* and *H*, Western blot examining GRIN1 expression using protein extracts of cortical neurons harvested at Day 45. N = 3 independent differentiations. *I*, confocal images of mature neurons at Day 45 stained for CAMKII and MAP2. Scale bar = 15 μm. Relative changes in CAMKII intensity of mature neurons (MAP2+) were quantified in (*J*) at different exposure doses. N ≥ 100 neurons from two independent differentiations. *K* and *L*, Western blot examining CAMKII expression using protein extracts of cortical neurons harvested at Day 45. N = 3 independent differentiation. *M*, typical wide-field images of mature neurons stained with live cell calcium indicator Fluo-4. Scale bar = 25 μm. *N*, relative changes in Fluo-4 intensity. N ≥ 60 neurons from three independent differentiations. Data = mean ± SE. N.S., not significant; ∗*p* < 0.05, ∗∗*p* < 0.01 and ∗∗∗*p* < 0.001.

Synapses are responsible for transmitting signals between neurons; and synapse loss is one of the key pathological features of AD (58, 59). We first characterized changes in synapses by evaluating the synaptic density of differentiated (Day 45) neurons co-stained for Synapsin1 (pre-synaptic marker) and HOMER1 (post-synaptic marker). Typical confocal images of co-stained neurites are shown in Figure 4*A*. We used SynQuant (60) to identify synapses along neurites with results summarized for the pre-synapses (Fig. 4*B*, left), post-synapses (Fig. 4*B*, middle) as well as synaptic pairs (Fig. 4*B*, right). Significant decreases were observed in pre-synaptic, post-synaptic, and synaptic pair density after 50 ppb Pb exposure. In cultures exposed to 15 ppb Pb, only the post-synaptic density showed a significant decrease. Conversely, the overall expression of Synapsin1 seems to increase over the neurites. Furthermore, protein expression of Synapsin1, HOMER1 and MAP2 were examined *via* Western blot using GAPDH as control (Fig. 4*C*). A decrease in HOMER1 expression was observed agreeing with the lower density of post-synapses as summarized in Figure 4*D* (left). Synapsin1 expression, however, increased with Pb treatments (Fig. 4*D*, middle) while MAP2 expression remained unchanged (Fig. 4*D* right). The discrepancy between Synapsin1 expression level and post-synaptic density suggests potentially altered protein transportation of Synapsin1 from the soma/axon to synapses. A closer examination of the post synaptic compartment co-stained for PSD95 and GRIN1 (Fig. 4*E*) further verified our findings. Despite reduced synaptic density, GRIN1 expression was significantly elevated in PSD95+ post-synaptic compartments in cultures exposed to 15 ppb Pb (Fig. 4*F*). Western blot further verified the increase in GRIN1 expression as shown in (Fig. 4, *E* and *F*), which is consistent with our transcriptomic findings (see Fig. 3*D*).

CaMKII is known to play a role in modulating calcium homeostasis (61) and was thus assayed *via* immunofluorescence. Confocal images of neurons stained for CaMKII are shown in Figure 4*I*, with their relative changes quantified in Figure 4*J*. The intensity of CaMKII staining of a single neuron was measured; and the analysis showed significant increases in CaMKII expression (>60%) at both 15 and 50 ppb Pb where the finding was further validated by Western blot summarized in Figure 4, *K* and *L*.

We used Fluo-4, a commonly used single-wavelength calcium indicator (62, 63), to further evaluate changes in intracellular calcium. Typical images of Fluo-4-stained neurons are shown in Figure 4, *M*, and *N* summarizes the relative changes in Fluo-4 intensity after Pb exposure. Noticeably, mature neurons showed significantly elevated Fluo-4 intensity with both 15 and 50 ppb Pb exposure. Intracellular calcium concentration is proportional to the fluorescent intensity of Fluo-4 assuming that prior Pb exposure does not affect the minimum and saturation maximum intensity of the Fluo-4 dye. Therefore, prior Pb exposure can alter calcium homeostasis in differentiated neurons.

### Developmental Pb exposure increases the risk of AD

To establish a connection between developmental Pb exposure and AD risk, we characterized changes in well-established pathological hallmarks of AD, namely, phosphorylated tau, beta-amyloid (Aβ) peptide, and tau aggregates formed by hyperphosphorylated tau (64, 65).

**Figure 5:**
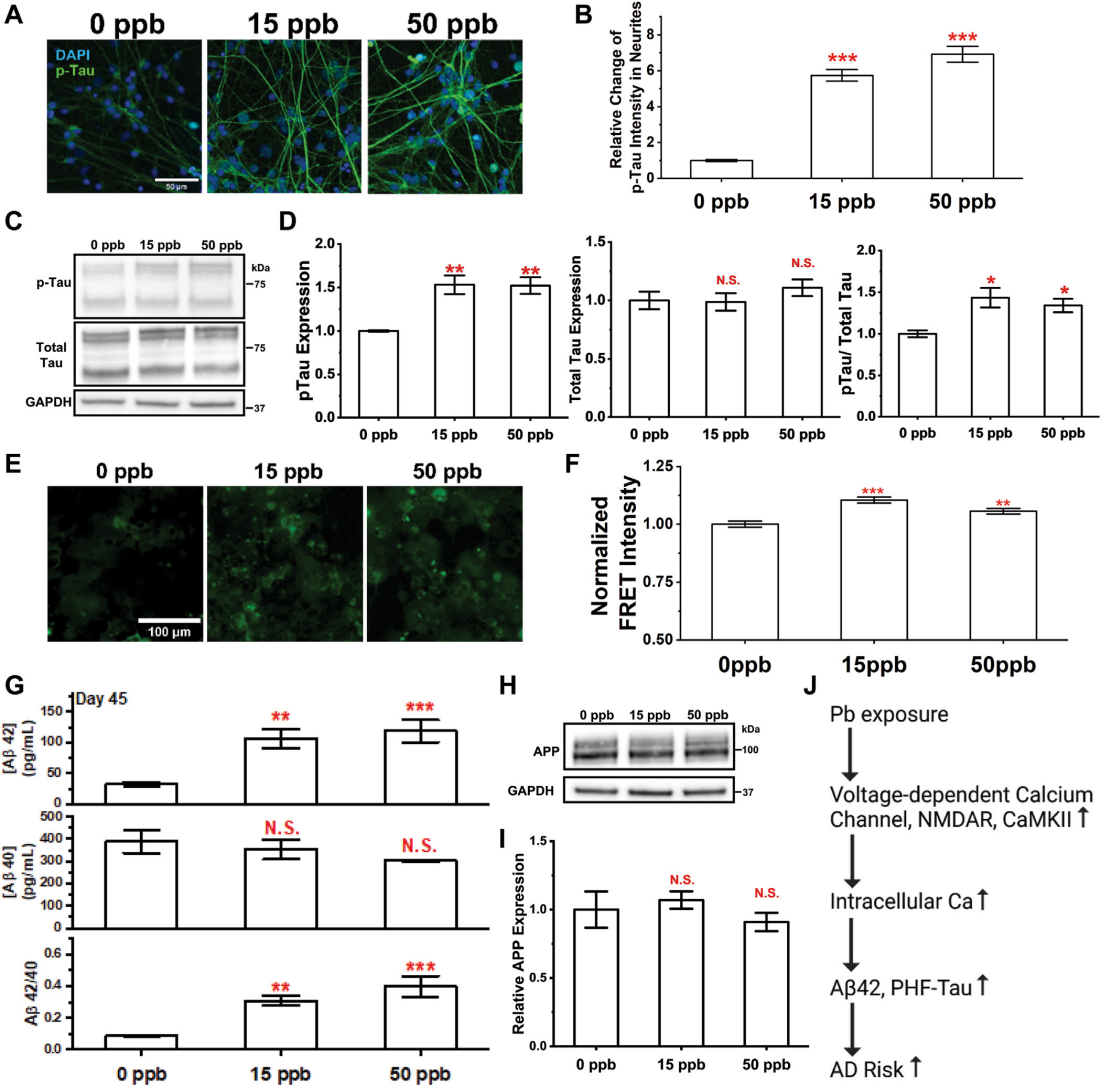
**Elevated AD pathological markers in differentiated neurons with developmental Pb exposure.***A*, confocal images of mature neurons at Day 45 stained with p-Tau (pThr181-Tau) and DAPI. Scale bar = 50 μm. The relative change of pThr181-tau in neurites was quantified in (*B*). N ≥ 60 neurites from two independent differentiations. *C* and *D*, p-Tau and total Tau expression were examined *via* Western blot using protein extracts of cortical neurons harvested at Day 45. N = 3 independent differentiations. *E*, representative FRET images of HEK293T tau biosensor cells treated with culture medium recovered from Pb exposed mature neuron culture at Day 45. *F*, the FRET intensity of HEK293T tau biosensor cells was quantified and normalized to the unexposed control. N = 8 medium samples harvested from four independent differentiations. The concentrations of Aβ_42_ (*G-top*) and Aβ_40_ (*G-middle*) in culture medium harvested at Day 45 were quantified *via* ELISA assays. Ratio of Aβ_42_/Aβ_40_ (*G-bottom*) was calculated based on each sample. N = 8 medium samples harvested from four independent differentiations. *H* and *I*, APP expression of cortical neurons harvested at Day 45 were detected and quantified *via* Western blot. N = 3 independent differentiations. Information of APP and CAMKII was extracted from the same blot, and (*H*) thus shared the same GAPDH control as 4K. *J*, illustration of hypothesized molecular pathway altered by developmental Pb exposure conferring increased AD risk. Data = mean ± SE. N.S., not significant; ∗*p* < 0.05, ∗∗*p* < 0.01, and ∗∗∗*p* < 0.001.

Multiple phosphorylation events at different sites on the tau protein have been associated with AD onset. Among these, phosphorylation of threonine 181 (Thr 181) is the most abundant in the middle stages (stage III/IV) of AD (66). We thus quantified pThr181-tau levels in our Pb exposed neurons stained with an antibody specific for this marker. Figure 5*A* shows typical images of pThr181-tau in mature neurons, and intensity analysis showed significantly increased pThr181-tau levels in neurites as summarized in Figure 5*B*. Western blot was also performed to quantify changes in pThr181-Tau and total Tau expression as shown Figure 5, *C* and *D*. Tau proteins are known to oligomerize and migrate as multiple bands on SDS-PAGE as demonstrated in previous literature (67, 68), these bands are thus all accounted for in our analysis. A significant increase in cellular pThr181-Tau and [pThr181-Tau]/[total Tau] was observed while total Tau expression remains unchanged. Although the general trend follows the observations in immunostaining, the magnitude of changes was much smaller in Western blot analysis. The discrepancy can be potentially attributed to the spatial preference of pThr181-Tau in dystrophic neurites.

As we observed significant increases in the levels of pThr181-tau, we utilized a biosensor reporter cell line to quantify changes in tau aggregates in the culture medium. This reporter cell line was developed by Hitt *et al* based on the expression of a truncated tau variant (K18-P301S) fused to two fluorescent proteins that constitute a FRET pair (69, 70). Culture medium harvested from mature neurons on Day 45 was added to the reporter cells and the integrated FRET intensity was recorded as a measure of the amount of intracellular tau aggregates formed *via* a seeding process. Figure 5*E* shows representative images of reporter cells visualized at a FRET setting. The normalized FRET intensity, calculated as FRET intensity/donor intensity, is summarized in Figure 5*F* (see Fig. S5 for negative control (fresh medium) and positive control (fresh medium with 50 nM of preformed fibrils prepared from recombinant tau (PHF-tau))). The results collectively suggest that developmental Pb exposure increases levels of tau seeds secreted in the medium by ∼ 10% and 6% for 15 and 50 ppb Pb-exposed samples, respectively.

We further quantified the amount of Aβ isoforms secreted in the culture medium by ELISA assays. At Day 45, we observed significant increases in Aβ_42_ concentrations in the culture medium harvested from neurons exposed to 15 and 50 ppb Pb, while Aβ_40_ concentrations remained almost unchanged as shown in Figure 5*E* top and middle. The Aβ42/40 ratio was increased significantly by ∼2.4 and ∼3.4 times at 15 and 50 ppb, respectively (see Fig. 5*E* bottom). The expression of amyloid beta precursor protein (APP) was quantified *via* Western blot showing no significant changes after Pb treatment (Fig. 5, *H* and *I*). Secretases associated with Aβ processing such as β- and γ-secretase also showed no significant alterations in our RNA-seq analysis.

## Discussion

Developmental Pb exposure is suggested as a potential environmental trigger for neurodegenerative diseases based on findings from human and animal studies. For example, a retrospective human study shows an association between prenatal Pb exposure and altered genes/enzymes that are implicated in amyloid plaque formation (71). Higher dose adult Pb exposure has been identified as an AD risk factor (72, 73), which suggests that cumulative lifetime Pb exposure is associated with accelerated declines in cognition and dementia (74). Studies using non-human primate, rodent, and zebrafish models also show that early-life Pb exposure results in transcriptional and histopathological changes related to neurodegeneration and pathologies related to AD in adult brains (75, 76, 77, 78). However, the molecular mechanism contributing to increased AD risk arising from developmental Pb exposure remains elusive.

We chose to work with cortical neurons derived from hiPSC since existing animal models do not necessarily capture the complexity of AD pathology or etiology. Currently, 15 ppb is the maximum Pb concentration allowed in drinking water set by US EPA (47). The median blood lead level of children in the United States between the ages of 1 to 5 years was determined as 0.6 μg/dl (6 ppb) in 2017 to 2018 which dropped 96% from the previous value of 15 μg/dl (150 ppb) in 1976 to 1980 (79). We thus chose to work with 15 and 50 ppb Pb due to its prevalence in the population. NPCs can serve as precursor cells for differentiation into various neuron subtypes, including cortical neurons and supporting cells (80), and they are abundant in both fetal and infant brains (81, 82). We thus set the exposure window during the NPC stage to mimic prenatal exposure, as well as, exposure in infants, *via* Pb in breastmilk (83).

NPCs with exposure to environmentally relevant levels of Pb differentiate into cortical neurons with no significant differences in neuron composition or nuclear morphology compared to the untreated control detected. The differentiating neurons, however, have increased neurite lengths and branch numbers at an immature stage, whereas these differences diminish at maturation. These data are consistent with previous studies using human embryonic cells (hESCs) exposed to Pb at 800 to 3800 ppb (84), and PC12 cells exposed to 50 ppb Pb (85) both of which showed increases in neurite length after Pb exposure. Meanwhile, we also observed changes in synaptic density. Pb exposure reduced the synaptic density for the pre-synapse, post-synapse as well as synaptic pairs. Loss of post-synaptic density is commonly observed in mouse models with AD mutations (86, 87, 88).

Epigenetic analysis of immature and mature neurons suggested decreases in repressive markers of immature neurons. The trend persists with neuron maturation for 5 mC but not for the selected bivalent markers, namely, H3K4me3 and H3K27me3. The loss of repressive markers after Pb exposure has been previously reported in various cell culture models (*e.g.*, SH-SY5Y and HEK293T cells) (39, 40, 54), animal models (*e.g.*, mouse and zebrafish) (38, 89) and a human cohort study (90); and was postulated to originate from altered activities of epigenetic enzymes (38, 91). The observed changes in Pb-exposed cortical neurons cannot be fully accounted for by the alterations in epigenetic enzymes observed in our transcriptomic analysis. Instead, cells seem to make attempts to normalize the acquired changes by modulating epigenetic enzyme levels. For example, although 5 mC levels are persistently reduced, we observed significant decreases in *TET1* and *TET2*, both of which are responsible for reducing methylation levels in cells.

The persistent 5 mC changes observed in Pb-exposed neurons led us to postulate that molecular pathways are compromised in mature neurons after developmental Pb exposure. Our RNA-seq analysis suggested that cellular responses to Ca were the top altered biological processes. Ca plays an essential role in neuronal function as a charge carrier and an intracellular messenger (92). Calcium signaling is involved in various developmental processes and plays a key role in apoptosis, neurotransmitter release, and membrane excitability (93, 94). Pb resembles Ca in both valency and structure and thus has a similar capacity for binding to Ca-specific targets. A closer examination of DEGs suggests major changes in NMDAR, CaMKII, and calcium channels, which we further verified in our results *via* immunostaining and calcium imaging. Neurons with developmental Pb exposure have increased NMDAR, CaMKII, and intracellular Ca concentrations. This finding is consistent with population studies suggesting elevated intracellular Ca concentrations in long-term Pb-exposed workers (95). Elevated CaMKII expression and activity were also previously reported in rat brains after prolonged (10 days) exposure to 1500 ppm Pb (96). Impaired NMDA receptor complex has been previously reported in a rat model with 1 μM of Pb exposure during synaptogenesis, and the resulting primary neurons showed significant upregulation in NR1 associated with PSD95 (97).

It Is also noteworthy that perturbations of NMDAR (98, 99) and CaMKII (100) are implicated in AD onset and progression. A detailed analysis of AD-related genes shows clear distinctions between unexposed and Pb-treated neuron samples (see Fig. 3*H*). We thus proceeded to evaluate changes in AD hallmarks, namely, p-tau, fibrillar tau, and Aβ42/40. We observed increases in all three markers in mature neurons unambiguously suggesting that a developmental Pb exposure can lead to an elevated risk of AD. The changes in tau pathology can be partially accounted for by increases in CaMKII expression and activity, which can catalyze the phosphorylation of tau proteins to promote the formation of aggregates (101, 102, 103, 104). Elevated tau phosphorylation was also reported in neurons derived from iPSCs of sporadic AD patients (105) and an isogenic line carrying the APP D679H mutation (106). The Aβ peptide arises from sequential cleavages of the amyloid precursor protein (APP) by β- and γ-secretase (107, 108). The activities of β-secretase (also known as BACE1) and γ-secretase are modulated by intracellular calcium concentrations (109, 110, 111), and an increase of these APP processing activities could contribute to the elevation of Aβ42/40 observed in this work. Remarkably, neurons derived from familial patients with AD, particularly with PSEN1 and PSEN2 mutations, have elevated levels of Aβ42 (112) and an increased Aβ42/40 ratio (113, 114, 115), similar to our observations.

Based on our collective findings, we have formulated a plausible mechanism accounting for increased AD risk after developmental Pb exposure as illustrated in Figure 5*F*. Specifically, developmental Pb exposure results in elevated expression of calcium channels (*e.g.*, NMDAR) and associated enzymes (CaMKII) to compensate for the binding and antagonism of these Ca-related effectors by Pb—compensatory effects that persist through neuronal maturation. After Pb is withdrawn and maturation is complete, the expression of these proteins is not completely restored, mostly likely due to the altered epigenetic landscape. As a result, maturing neurons adopt an altered Ca homeostasis state favoring higher intracellular calcium concentrations, thus impacting the activity of Ca/calmodulin-dependent kinase, such as CaMKII, and APP-processing secretase, such as BACE1, in turn leading to the accumulation of protein variants with a high potential to form aggregates, including p-tau and Aβ42. Over the long term, the accumulation of such seeds can result in the formation of pathological inclusions (*e.g.*, tau neurofibrillary tangles and Aβ plaques) that are manifested in patients with AD and commonly conceived as key neuropathological signs of AD.

Our findings provide the first evidence linking developmental Pb exposure with elevated AD risk using cortical neuronal cultures derived from hiPSC. Neurons exposed to Pb at the progenitor stage have increased intracellular calcium concentrations and neurite lengths along with elevated AD molecular markers. Persistent DNA methylation changes were also observed in mature neurons, providing a potential mechanism for transmitting long-term AD risk. A plausible mechanism is proposed connecting developmental Pb exposure and AD risk.

## Experimental procedures

### hiPSC cell culturing and cortical neuron differentiation

We cultured hiPSCs (HPSI0114i-kolf_2) to generate mature cortical neurons following an established protocol (43). Specifically, we started by culturing hiPSC on tissue culture plates coated with Matrigel (Corning, 354277) using a StemFlex medium (ThermoFisher, A3349401) at 37°C with 5% CO2. hiPSCs were then replated into an AggreWell 800 Plate (Stemcell Technologies, 34811) with a density of ∼10,000 cells per microwell to form embryoid bodies (EB). EBs were then maintained in a SMADi neural induction kit (Stemcell Technologies, 08581) for 5 days. After that, we replated EB spheres into a Matrigel-coated plate using wide-bore tips to form neural rosettes. Rosettes were maintained for another 7 days and were selected using a neural rosette selection reagent (Stemcell Technologies, 05832) followed by replating to generate NPCs in a neural progenitor medium (Stemcell Technologies, 05833). The identity of NPCs was confirmed with immunostaining of PAX6 (Invitrogen, 21103049) and FOXG1 (Abcam, ab18259) as shown in Fig. S1.

After that, we differentiated NPCs into cortical neurons using a forebrain neuron differentiation kit (Stemcell Technologies, 08600). The differentiation starting date was counted as Day 0 as shown in Figure 1*A*. Pb stock solutions were prepared as previously described (39) and diluted into selected concentrations (15 and 50 ppb) in the culture medium. Pb doses were selected based on the current US EPA regulation standard (15 ppb) and thus a high likelihood of exposure during lifetime. Pb solutions were spiked into cell culture medium on Day −2 for 48 h and rinsed out on Day 0 *via* three washes by 1 × DPBS (Gibco, 14190144). After that, we switched the culture medium to the forebrain neuron differentiation medium (Stemcell Technologies, 08605) for 8 days then the maturation medium until the time of assessments.

### Immunostaining

We used immunocytochemistry to characterize neurons at different stages. Cells were fixed and stained following protocols as we described in our previous work (39, 40, 52, 116). Primary antibodies of TBR2 (Abcam, ab183991), TBR1 (Abcam, ab183032), VGLUT1 (SySy, 135302), GFAP (Cell Signaling Technology, 3670S), MAP2 (SySy, 188004), TUJ1 (SySy, 302304), Synapsin 1 (SySy, 106011), Homer 1 (SySy, 106003), 5-mC (Active Motif, 61479), H3K4me3 (Abcam, ab8580), H3K27me3 (Abcam, ab192985), GRIN1 (Invitrogen, PA3102), PSD95 (Invitrogen, MA1046), CaMKII (Santa Cruz Biotechnology, sc-5306) and pThr181-Tau (Invitrogen, MN1050) were used. Anti-mouse Alexa-488 (Invitrogen, A11001), anti-mouse Alexa-568 (Invitrogen, A11004), anti-rabbit Alexa-488 (Invitrogen, A11008), anti-rabbit Alexa-568 (Abcam, ab175471), anti-guinea-pig Alexa-594 (Invitrogen, A11076) and anti-guinea-pig Alexa-647 (Invitrogen, A21450) were used as secondaries. DAPI (Sigma, D9542) was used to stain the nucleus.

### Western blotting

Cell pellets of hiPSC-derived cortical neurons were lysed in 1 × RIPA buffer (150 mM NaCl, 50 mM Tris, pH 8.0, 0.1% SDS, 1% NP-40, 0.5% sodium deoxycholate) containing protease and phosphatase inhibitor mixture (ThermoScientific, 78441) at 4 °C for 30 min. The lysate was then centrifuged at 12,000*g* for 20 min at 4 °C; and the resulting supernatant was collected. The protein concentration in the supernatant was quantified *via* a BCA protein assay kit (ThermoScientific, 23252). ∼15 μg of protein was loaded in each well of Bis-Tris gels (Invitrogen, NW04120BOX) followed by electrophoresis in 1× Bolt MES buffer (Invitrogen, B0002). Proteins were then transferred to the nitrocellulose membrane (Invitrogen, IB23002) *via* an iBlot2 gel transfer device (ThermoScientific, IB21001). Membranes were then blocked in 1× TBST buffer (20 mM Tris, pH 7.5, 150 mM NaCl and 0.05% Tween 20) with 5% milk powder overnight at 4 °C. Hybridization with primary antibodies was carried over overnight at 4 °C. Membranes were then rinsed with 1× TBST buffer three times at room temperature followed by hybridization with horseradish peroxidase (HRP)-conjugated secondary antibody. After that, membranes were rinsed again in 1× TBST followed by incubation with enhanced chemiluminescence substrates (Bio-Rad, 1705060) at room temperature for 5 min. Membranes were immediately visualized using a ChemiDoc Imaging System (Bio-Rad) followed by quantification using Image J (NIH). GAPDH were used as control.

In addition to the primary antibodies mentioned in the immunostaining section, primary antibodies used here include GAPDH (Invitrogen, MA5-15738), Tau (Invitrogen, PIPA595648), APP (Abcam, ab32136), and Histone H3 (Abcam, AB1791–1001). HRP anti-mouse (Active Motif, 15014), HRP anti-rabbit (Invitrogen, 65–6120), and HRP anti-chicken (Invitrogen, A16054) were used as secondary.

### Fluorescence microscopy

A high-content imaging microscope (Molecular Device, ImageXpress Micro Confocal) was used to collect fluorescent and DIC images using Nikon Plan Apo 20 × /0.75 NA or 60 × /1.2 NA Water objective. Confocal images were collected with a Z step-size of 1 μm.

### Calcium imaging

A Fluo-4 Calcium detection kit (Invitrogen, F10471) was used to detect intracellular Ca concentrations. We followed the manufacturer’s protocol to stain the cells. Briefly, the Fluo-4 loading solution was added to the cortical neuron medium at the time of assessment. We then exchanged the culture medium into Fluo-4-free maturation medium after ∼ 45 min incubation. Cells were imaged immediately using ImageXpress Micro Confocal (Molecular Device) with Nikon Plan Apo 20 × /0.75 NA objective in an environment-controlled chamber.

### RNA-sequencing

The hiPSC-derived neurons from three independent differentiations were pelleted *via* centrifugation at 500 rcf for 5 min followed by total RNA extraction using a PureLink RNA Mini Kit (Invitrogen, 12183018A). The concentrations of extracted total RNA were measured *via* a Nanodrop UV-Vis spectrometer (ThermoFisher) and subsequently stored at −80 °C prior to sequencing. The quality of RNA was verified by Agilent Bioanalyzer 2100 system before sequencing. Only RNA with RNA integrity number (RIN) scores higher than 6.0 (characterized by an Agilent Bioanalyzer 2100 system) proceeded to library preparation followed by sequencing using HiSeq (Illumina) at Novogene Inc. All sequencing was performed with an average sequencing depth of 6G raw data. The Homo Sapiens genome (GRCh38/hg38) was used as a reference. EdgeR was used to determine DEGs using a *p*-value of 0.05. Pathway analysis was performed using GO. Three samples from each group were sequenced and analyzed.

### Tau seeds transfection and FRET imaging of tau-biosensor

Cell culture medium was harvested and stored at −80 °C until analysis. An HEK293T tau biosensor line was used to detect and quantify tau seeds in a culture medium (69, 70). Briefly, this reporter cell line has stably integrated DNA encoding tau repeat-domain (RD) variants with the P301S mutation fused to mClover3 or mCerulean3, which aggregate in the presence of internalized tau seeds to produce a FRET signal positively correlated to the concentration of fibrillar tau. Reporter cells were seeded into a 96-well TC plate at a confluency of 40% 24 h prior to the addition of seeds or conditioned media. Lipofectamine 3000 (Invitrogen, L3000015) was used to stimulate the uptake of pre-existing tau seeds as described previously (69). The transfection mixture for each well of 96-well plate (∼1.5 × 10^4^ cells) contained 40 μl of harvested neuronal medium, 1.25 μl of Lipofectamine 3000, and 10 μl of Opti-MEM (Gibco, 31985070). Cells were incubated with a transfection mixture for 24 h before imaging.

Human tau 2N4R was purified from BL21(DE3) *E. coli* cells transformed with the plasmid pRK172-2N4R (kindly provided by Dr David Eliezer, Weill Cornell Medicine) (117). The cells were grown in LB media supplemented with ampicillin (100 μg/ml), and protein expression was induced by the addition of isopropyl β-d-1-thiogalactopyranoside (IPTG, 1 mM) for 4 h at 37 °C. At the end of the induction period, the cells were pelleted by centrifugation at 6000*g* for 15 min at 4 °C, resuspended in lysis buffer (20 mM MES, 400 mM NaCl, 0.2 mM MgCl_2_, 1 mM EGTA, protease inhibitor cocktail (P8340, Sigma Aldrich), 0.25 mg/ml lysozyme, and 1 μg/ml DNase I, pH 6.8), and lysed with a French pressure cell disruptor at 4 °C. The lysate was boiled for 20 min, denatured proteins were pelleted by centrifugation at 30,000*g* for 30 min at 4 °C, and the supernatant was dialyzed overnight against a cation exchange buffer (20 mM MES, 50 mM NaCl, 1 mM MgCl_2_, 1 mM EGTA, 2 mM DTT, 0.1 mM PMSF, pH 6.8). The dialysate was loaded onto a HiPrep SP HP column, and proteins were eluted with a linear gradient ranging from 50 mM to 1 M NaCl. Fractions containing tau isoform 2N4R (as determined *via* SDS-PAGE with Coomassie blue staining) were pooled, and the resulting protein solution was dialyzed against PBS (pH 7.4) and stored at −80 °C.

For FRET imaging, we used a high-content imaging microscope (Molecular Device, ImageXpress Micro Confocal) with a Nikon Plan Apo 20 ×/0.75 NA objective. The donor was excited at 395/25 nm. The emission light was filtered through a 460/50 nm emission filter and a 536/40 nm emission filter for donor and FRET signals, respectively.

### ELISA assay for β-amyloid and global DNA methylation quantification

Aβ_40_ (Invitrogen, KHB3481) and Aβ_42_ (Invitrogen, KHB3441) specific ELISA assay kit was used to quantify the concentration of two isoforms of β-amyloid secreted by neurons. 25 μl of neuronal culture medium was used for each well and the assay was performed following the manufacturer’s protocol. We used a global DNA methylation LINE-1 kit (Active Motif, 55,017) to quantify global DNA methylation level. Genomic DNA (gDNA) was extracted using a PureLink Genomic DNA Mini Kit (Invitrogen, K182001); and concentration was determined *via* NanoDrop UV-Vis spectrometer (ThermoFisher). 100 μg gDNA were applied to each well to determine their methylation level following the manufacturer’s protocol. All absorbance measurements were performed using a SpectraMax iD3 plate reader (Molecular Device).

### Data analysis and statistics

Fluorescence intensity and morphological features of epigenetic modifications were analyzed *via* a custom-built analysis pipeline in CellProfiler (Broad Institute) as we described previously (39, 52). Neurite analysis was performed using the Neurite Outgrowth Module embedded in MetaXpress software (Molecular Device). Other fluorescent intensities were quantified using ImageJ (NIH). SynQuant was used to identify and quantify synapses following a standard protocol (60). Relative expression of proteins was determined by quantifying the protein band intensity normalized to GAPDH intensity using ImageJ (NIH).

All results were reported as mean ± standard error (SE). Statistical analysis was performed using OriginPro 2021. Statistical differences in quantities were determined using one-way ANOVA followed by Fisher’s LSD for means comparison with *p-value < 0.05*.

## Data availability

The data that support the findings of this study are available from the corresponding author upon reasonable request.

## Supporting information

This article contains supporting information (118, 119, 120, 121, 122, 123, 124, 125, 126, 127, 128, 129, 130, 131, 132, 133, 134, 135, 136, 137, 138, 139, 140, 141, 142, 143, 144, 145, 146, 147, 148, 149, 150, 151, 152, 153, 154).

## Conflict of interest

The authors declare that they have no conflicts of interest with the contents of this article.
