# Supplementary material for: Developmental Pb exposure increases AD risk via altered intracellular Ca2+ homeostasis in hiPSC-derived cortical neurons
Source: J Biol Chem. 2023 Jul 7;299(8):105023. doi: 10.1016/j.jbc.2023.105023 (PMC10413359; doi:10.1016/j.jbc.2023.105023)
Supplement: Supporting information [file mmc1.pdf]

Developmental Pb exposure increases AD risk via altered intracellular Ca^{2+} homeostasis in hiPSC derived cortical neurons

Junkai Xie[1], Shichen Wu[1], Hailey Szadowski[2], Sehong Min[3], Yang Yang[3,5], Aaron B. Bowman[4,5], Jean-Christophe Rochet[3,5], Jennifer L Freeman[4,5,6], Chongli Yuan[1,4,6*]

1. Davidson School of Chemical Engineering, Purdue University, West Lafayette, IN, 47907
2. Agriculture and Biological Engineering, Purdue University, West Lafayette, IN, 47907
3. Department of Medicinal Chemistry and Molecular Pharmacy, Purdue University, West Lafayette, IN, 47907
4. Purdue Institute of Integrated Neuroscience, Purdue University, West Lafayette, IN, 47907
5. School of Health Sciences, Purdue University, West Lafayette, IN, 47907
6. Purdue Center of Cancer Research, West Lafayette, IN, 47907

*: To whom correspondence should be addressed. CY: cyuan@purdue.edu

Supporting Information

1. Supporting Tables
2. Supporting Figures
3. Supporting References

SUPPORTING TABLES

1. **Table S1**: Differentially Expressed Genes from Pb treatment (See Separate Excel File)
2. **Table S2**: DEGs associated with AD in **Figure 3H** (*: collected based on the IPA database)

**Genes in dataset**	**Association with AD***
STAR	Affects (118)
CHAT	Affects (119)
CALB1	Affects (120)
SYP	Affects (121, 122)
CAMK2B	Affects (123)
ACHE	Affects (124, 125)
PRKCB	Affects (126, 127)
SNRNP70	Affects (128)
BAX	Affects (124)
PRKCZ	Affects (123)
GRIN1	Affects (129, 130)
SNCA	Affects (131, 132)
VEGFA	Affects (133)
CHD5	Affects (133)
FAAH	Affects (134, 135)
BIN1	Affects (136)
GAP43	Affects (123, 137)
ABCA7	Affects (136)
SV2A	Affects (123)
SNAP25	Affects (133)
NFS1	Affects (128)
JUN	Affects (138)
BAG1	Affects (139, 140)
BCL2	Affects (141)
APBB1	Affects (142)
SMTN	Affects (133)
XPO7	Affects (128)
VPS35	Affects (143)
WDFY3	Affects (128)
NUP98	Affects (144)
LARP4	Affects (126)
SLC1A3	Affects (145)
SPARCL1	Affects (133)
MT-CO1	Affects (146)
SLC2A1	Affects (123)
CCNB1	Affects (147, 148)
NFE2L2	Affects (149)
RTN4R	Affects (150)
NTRK2	Affects (124, 133, 151, 152)
CDK1	Affects (147, 153)
NQO1	Affects (154)
WIF1	Affects (133)
NMNAT3	Affects (128)

PTPRZ1	Affects (128)

SUPPORTING FIGURES

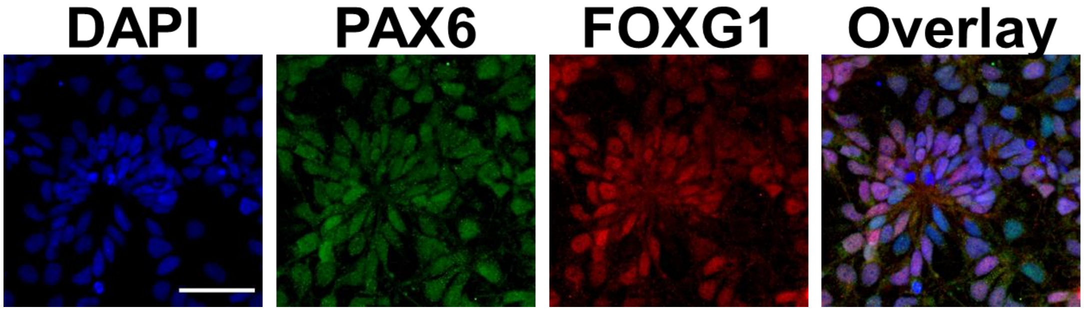

Fig. S1: Typical images of NPC stained for DAPI, PAX6 and FOXG1. Scale bar = 50 μm.

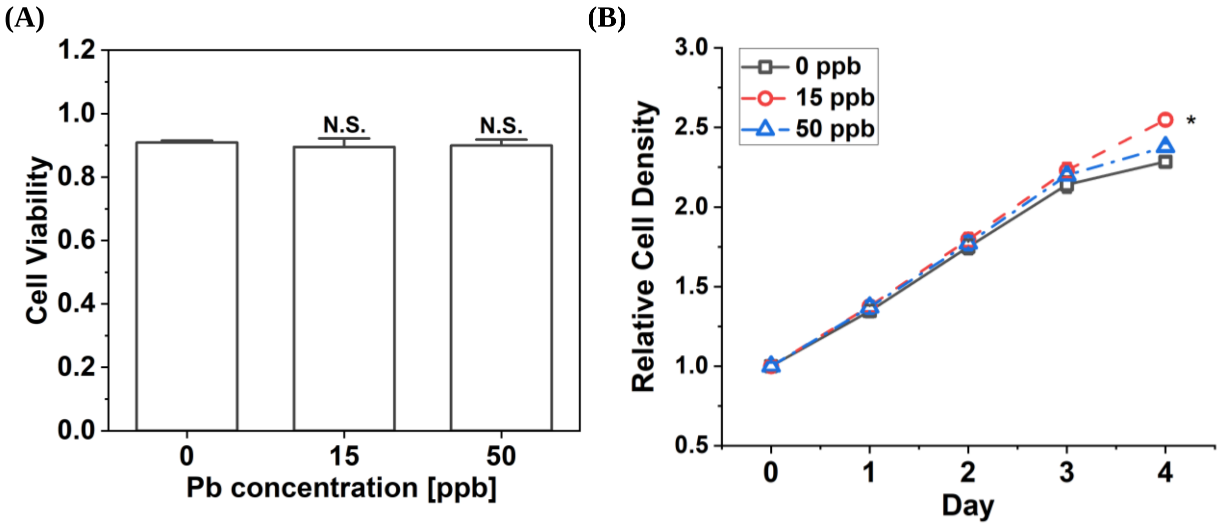

Fig. S2: **(A)** Cell viability of neural progenitor cells after Pb exposure. N = 5. **(B)** Relative cell density after treating with Pb of varying doses. Relative cell density was calculated by normalizing cell numbers normalized to that of Day. N = 17. Data = Mean ± S.E.. *: $p < 0.05$. and N.S.: not significant.

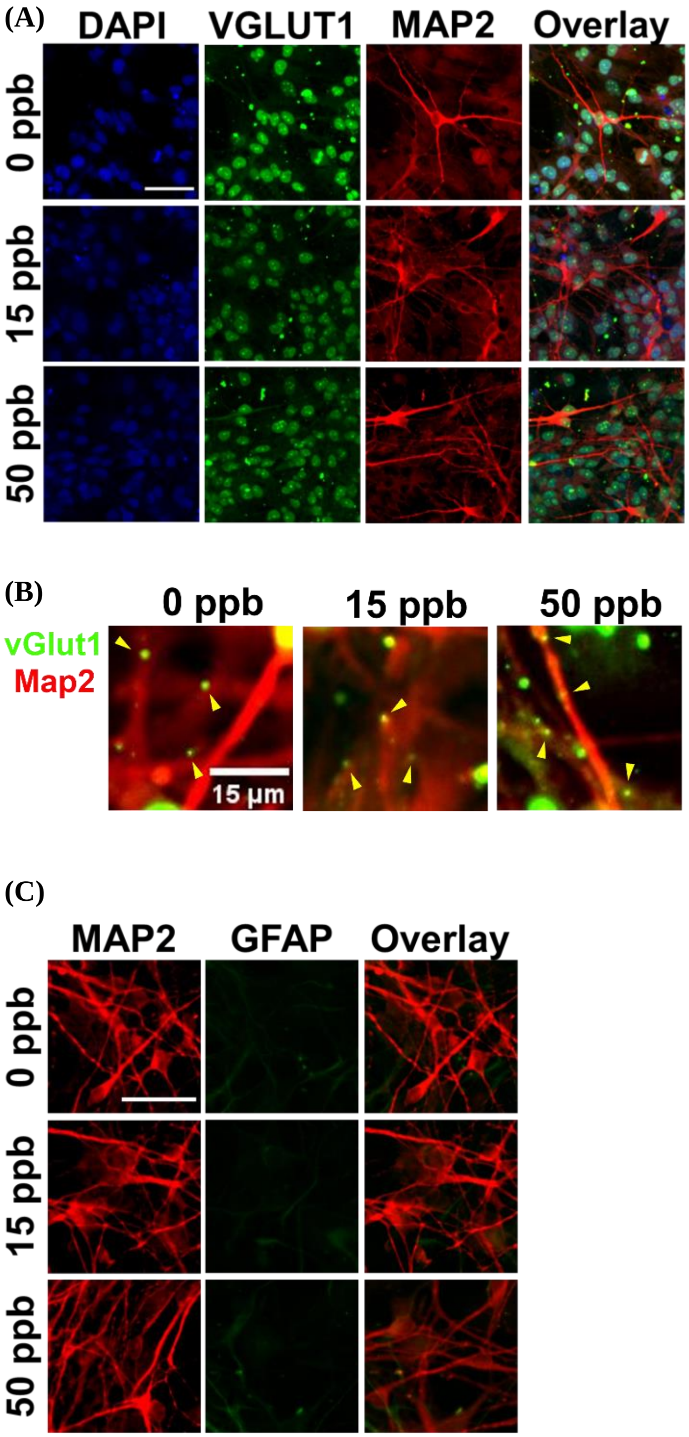

Fig. S3: **(A)** Representative wide-field images of mature neurons at day 35 stained for DAPI, VGLUT1 and MAP2. Scale bar = 50 µm. **(B)** VGLUT1+ synapses on mature neurons at day 35 indicated by yellow arrows. Scale bar = 15 µm. **(C)** Representative wide-field images of mature neurons at day 35 stained for MAP2 and GFAP. Scale bar = 50 µm.

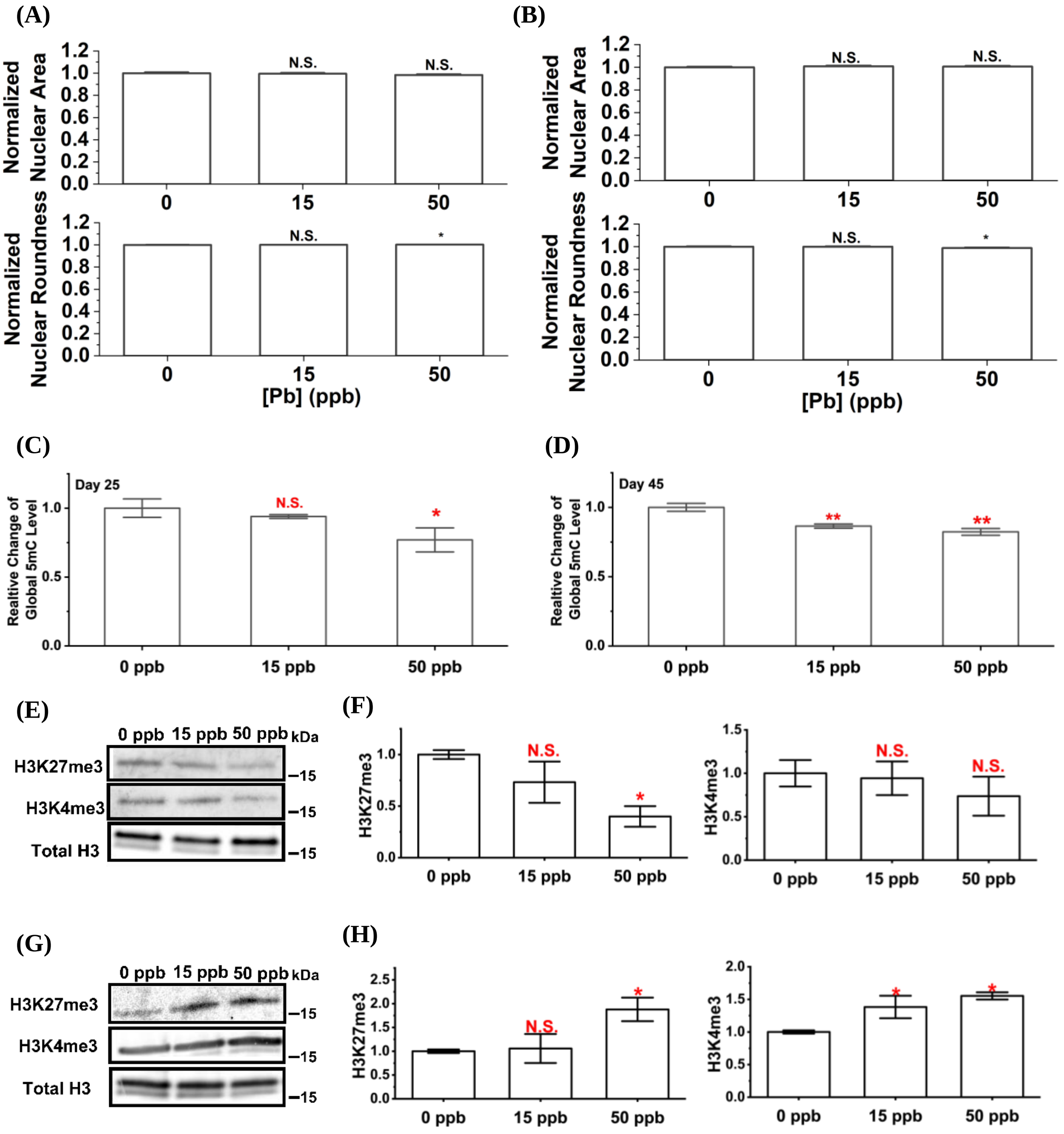

Fig. S4: Relative changes in nuclear area and roundness of (differentiating) neurons at **(A)** day 25 and **(B)** day 45. N = 6 independent differentiations. **(C-D)** Global DNA methylation level were examined via ELISA. N = 3 independent differentiations. The expression of H3K27me3 and H3K4me3 normalized to total H3 at **(E-F)** Day 25 and **(G-H)** Day 45 were quantified via western blot. N = 3 independent differentiations. Data = Mean ± S.E.. *: $p < 0.05$. and N.S.: not significant.

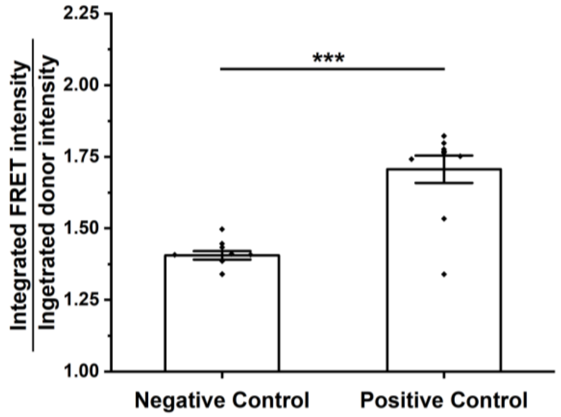

Fig. S5: Integrated FRET intensity to integrated donor intensity ratio of a negative control (transfection vehicle only) and positive control (50 nM of PHF-tau). N = 10 views from 2 biological replicates. Data = mean ± S.E. ***: $p < 0.001$.
